# Fingerprint Recognition Based on Molecular-Scale Conductance Response via Electrochemically Gated Quantum Tunnelling

**DOI:** 10.3390/s26092896

**Published:** 2026-05-05

**Authors:** Zifan Wang, Long Yi, Ga Zhang, Xufei Ma, Ye Tian, Bintian Zhang, Xu Liu, Longhua Tang

**Affiliations:** 1 State Key Laboratory of Extreme Photonics and Instrumentation, Interdisciplinary Center for Quantum Information, College of Optical Science and Engineering, Zhejiang University, Hangzhou 310027, China; zifanw@zju.edu.cn (Z.W.); gazhang@zju.edu.cn (G.Z.); maxufei@zju.edu.cn (X.M.); xuliu@zju.edu.cn (X.L.); 2 Nanhu Brain-Computer Interface Institute, Second Affiliated Hospital School of Medicine, Hangzhou 311100, China; 3 State Key Laboratory of Fluid Power and Mechatronic Systems, College of Mechanical Engineering, Zhejiang University, Hangzhou 310058, China; ye-tian@zju.edu.cn; 4 Shenzhen Key Laboratory of Precision Measurement and Early Warning Technology for Urban Environmental Health Risks, School of Environmental Science and Engineering, Southern University of Science and Technology, Shenzhen 518055, China; zhangbintian@sustech.edu.cn

**Keywords:** quantum tunneling, electrochemical gating, near-single-molecule detection, conductance fingerprints, molecular junctions

## Abstract

Molecular-scale detection based on quantum tunnelling is promising for molecular electronics and high-sensitivity analysis, owing to its sensitivity to molecular structure and energy levels. However, conventional two-electrode tunnelling measurements suffer from overlapping conductivity of different molecules, limiting molecular discrimination in complex systems. To address this, we propose an electrochemical-gate-controlled nanoscale tunnelling strategy that expands the two-electrode system to a three-electrode configuration via a tunable gate potential, enabling the differentiation of distinct molecules at near-single-molecule sensitivity. Scanning the gate potential under constant tunnelling bias modulates the alignment between molecular orbitals and the electrode Fermi level, altering the statistical characteristics of molecular tunnelling transport. Experimental results show that target molecules induce a bimodal distribution of tunnelling current (background and molecule-correlated channels), with the second peak exhibiting distinct gate potential dependence. Comparative analysis of ascorbic acid (AA), acetylcholine (ACh), and uric acid (UA) reveals unique trajectories of characteristic peaks with gate potential, forming an electrochemical gate response fingerprint. This gate-dependent conductance trajectory provides a novel statistical dimension for molecular recognition, enabling differentiation of distinct molecules.

## 1. Introduction

Near-single-molecule detection, an interdisciplinary field spanning nanoscience, analytical chemistry, and molecular electronics, enables direct nanoscale electrical detection of individual molecules, complementing bulk measurement methods [1]. Quantum-tunnelling-based detection has emerged as a research hotspot due to its ultra-high spatial resolution and sensitivity to changes in molecular structure/energy levels [1]. This phenomenon, where electrons traverse classical energy barriers, generates tunnelling currents sensitive to nanoscale structural variations and molecule–electrode interactions, forming the basis of high-sensitivity single-molecule probes [1]. In the past decades, significant advances in quantum tunnelling research platforms have enabled the construction of stable single-molecule junctions and the reliable analysis of conductance distributions [2,3]. These tools facilitate investigations of molecular electronic transport and extend to high-sensitivity chemical/biomolecular analysis [3,4].

However, tunneling sensors inherently exhibit limited molecular selectivity. When two molecules with comparable conductance characteristics enter the tunneling gap, they generate highly similar tunneling current responses. Thus, it is difficult to differentiate such molecules through tunneling current signals without the assistance of advanced analytical techniques. [1]. In addition, quantum tunnelling is extremely sensitive to the local environment. Any interference in the tunnelling gap can introduce noise and disturb the current signal, making it difficult to read out. This noise can come not only from contamination by other molecules but also from gap-distance fluctuations and the adsorption of molecules on the tunnelling electrode surface [4]. During sensing in the solution phase, the orientation of target molecules in the tunnelling gap [5,6], intermolecular interactions between molecules [7,8], and alignment with the electrode interface may lead to distinct conductance pathways for the same molecule [9]. These factors can broaden the tunnelling signals, making molecular identification difficult.

To solve these problems, surface modification has been developed to introduce selectivity into tunnelling junctions. Based on the material of the tunnelling nanoelectrode, different functional molecules can be modified on the electrode surface. Through this strategy, target molecules can either be bridged into the tunnelling junction through chemical bonds to form a single-molecule junction, allowing the conductance of one molecule to be measured and used to reveal its chemical and physical properties [10]. Alternatively, target molecules can be captured through intermolecular interactions, such as hydrogen bonding [11], π–π interactions [12], and supramolecular interactions [13], which is known as recognition tunnelling [14]. Recognition tunnelling can help fix target molecules in a more specific state within the tunnelling gap, while the recognition/anchoring terminal can improve electronic coupling between the electrode and target molecule, thereby improving the signal and reducing non-specific adsorption [14]. During functionalization, the modified molecules can also enhance the stability of the electrode surface [15]. This method has been widely applied to distinguish structurally similar molecules, such as nucleotides and amino acids [16,17,18].

In addition to surface modification, external physical fields can also be used to optimize the tunnelling pathway. For instance, introducing a “gate” mechanism is a promising approach to enhance controllability. In solution-based systems, electrochemical gating flexibly modulates the alignment between molecular orbitals and the electrode Fermi level by adjusting the electrode–solution interface potential, altering molecular-scale conductivity [19,20,21]. Prior studies have verified electrochemical gating’s ability to regulate conductance and transport mechanisms [22,23,24], but its application to molecular recognition via gate-dependent response trajectories remains underexplored.

Herein, we combine electrochemical gating with recognition tunnelling to overcome traditional dual-electrode limitations. Using neurotransmitters and structurally similar small molecules as models, we scan gate potentials to control orbital alignment, generating unique “conductance response fingerprints” that enhance discrimination of structurally similar compounds.

## 2. Materials and Methods

### 2.1. Reagents and Solution Preparation

Phosphate-buffered saline (PBS, 10 mM, pH 7.4) was purchased from Sigma-Aldrich (St. Louis, MO, USA). Dopamine hydrochloride (DA), ascorbic acid (AA), acetylcholine (ACh), and uric acid (UA) (all ≥98% purity, Sigma-Aldrich) were dissolved in PBS (10 mM, pH 7.4). PBS buffer was degassed with high-purity argon (Ar, ≥99.9%) for 30 min to remove dissolved oxygen. DA solution (10 µM) was used for tunnelling detection; AA, ACh, and UA (each 100 nM) were used for control experiments. All experiments were performed at room temperature.

### 2.2. Fabrication of Nanoscale Quantum Mechanical Tunnelling Probes

Quantum mechanical tunnelling probes were fabricated according to our previously reported method [25]. Briefly, dual-channel quartz nanopipettes were fabricated using a laser-assisted capillary puller (P-2000, Sutter Instrument, Novato, CA, USA) with the following conditions and parameters: 25 °C, Heat 850, Fil 4, Vel 30, Del 160, Pul 100, yielding a dual-nanopore tip (≈30 nm diameter). Carbon nanoelectrodes were formed by thermal decomposition of butane gas (≥99.9%) inside the pipette, followed by electrochemical etching (+1.2 V vs. Ag/AgCl in 0.1 M KCl) to clean the electrodes. Gold electrodes were fabricated via two-step electrodeposition in 0.05 M HAuCl_4_ solution: initial plating (−0.8 V vs. Ag/AgCl, 30 s) followed by constant-current feedback (2 nA) until the electrode gap reached the tunnelling range. Probes were rinsed with ultrapure water and stored at 4 °C before use. Prior to experiments, probes were immersed in a 0.5 mM solution containing 4(5)-(2-mercaptoethyl)-1H-imidazole-2-carboxamide (ICA) for 2 h and then rinsed with PBS to remove unbound ICA. ICA functionalization constrains molecular orientation via specific hydrogen bonding, minimizing the impact of conformational fluctuations on orbital alignment and ensuring stable conductance signals.

### 2.3. Electrochemical-Gated Tunnelling Measurement System

Tunnelling current was measured using a dual-channel patch-clamp amplifier (MultiClamp 700B, Molecular Devices LLC., San Jose, CA, USA) and a high-speed digitizer (Digidata 1550B, Molecular Devices, USA) at a 100 kHz sampling frequency, with a 10 kHz low-pass Bessel filter. The two tunnelling electrodes (WE_1_ and WE_2_) were connected to the amplifier’s channels, sharing an Ag/AgCl reference electrode. The system was grounded and placed in a Faraday shielding chamber to minimize electromagnetic interference. Electrode potentials were defined as $V_{WE1}\;=\;V_{g}\;$ (gate potential, vs. Ag/AgCl) and $V_{WE2}\;=\;V_{g}+\;V_{b}$ ($V_{b}$, tunnelling bias). $V_{g}\;$ was scanned, while $V_{b}$ remained constant.

### 2.4. Gate Potential Scanning Procedure

The quantum tunnelling probes and the reference electrode were placed in a 50 μL microfluidic cell containing 10 mM PBS solution (pH = 7.4). $V_{g}\;$ was scanned from −0.2 V to +0.4 V vs. Ag/AgCl at 200 V/s, with $V_{b}$ fixed at −0.05 V. Each experiment recorded 1000 consecutive scans, with ≥20 independent replicates per solution. After each measurement, electrodes were rinsed with 5% (*w*/*w*) ammonium bicarbonate for 5 min, and the cell was refilled with fresh PBS for 10 min to restore baseline current before experiment. All data was collected and transferred through Clampfit. The heat map plot and bimodal Gaussian distribution fitting were analyzed by a custom-written code.

## 3. Results

### 3.1. Electrochemically Gated Tunnelling Measurement

**Figure 1:**
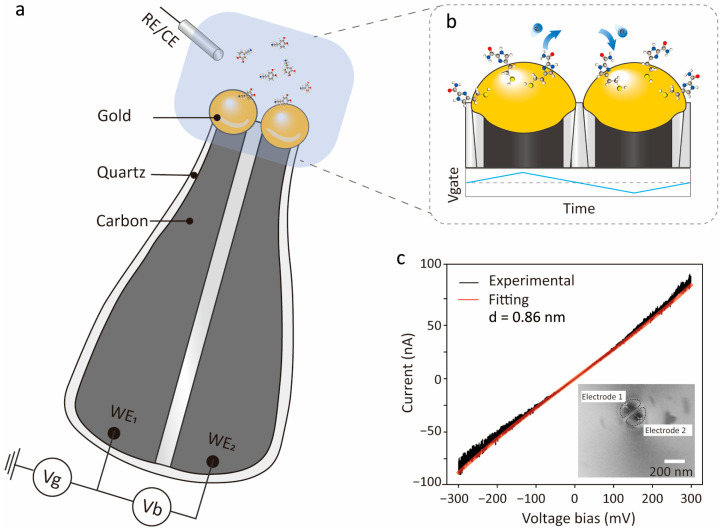
Structure of the electrochemically gated tunnelling electrode and electrical characterization of the device. (**a**) Schematic of the tunnelling electrode device. Two gold tunnelling electrodes served as working electrodes WE_1_ and WE_2_, while an Ag/AgCl electrode was used as the reference electrode (RE) to enable electrochemical gate control. (**b**) Schematic of the tunnelling region. The electrode surface was functionalized with the thiol-anchored molecule 4(5)-(2-mercaptoethyl)-1H-imidazole-2-carboxamide (ICA) to form a molecular recognition interface. (**c**) Representative current–voltage (I–V) response (black curve) of the tunnelling electrode. The experimental data (black) were well fitted by the Simmons tunnelling model (red curve), giving an estimated gap distance of d = 0.86 nm. The inset shows an optical micrograph of the electrode tip, demonstrating the actual morphology of the molecule.

To investigate the regulatory effect of electrochemical gating on single-molecule tunnelling, we constructed a high-sensitivity electrochemical tunnelling system based on a dual-pore nanoprobe. As shown in Figure 1a, the probe was fabricated from a dual-channel quartz nanopipette with carbon-gold electrodes at the tip (nanoscale spacing) to form a stable tunnelling junction in the electrolyte, ensuring reliable molecular-scale measurements. Figure 1b details the dual-electrode tip functionalized with ICA molecules, which form stable Au-S bonds with gold electrodes to construct a molecular recognition interface near the nanogap. Figure 1c shows the typical I–V curve, which is well-fitted by the Simmons model, estimating an interelectrode gap of ≈0.86 nm (ideal for molecular-scale tunnelling detection, 0.5–2 nm). The inset confirms the electrode tip morphology, verifying successful device fabrication.

**Figure 2:**
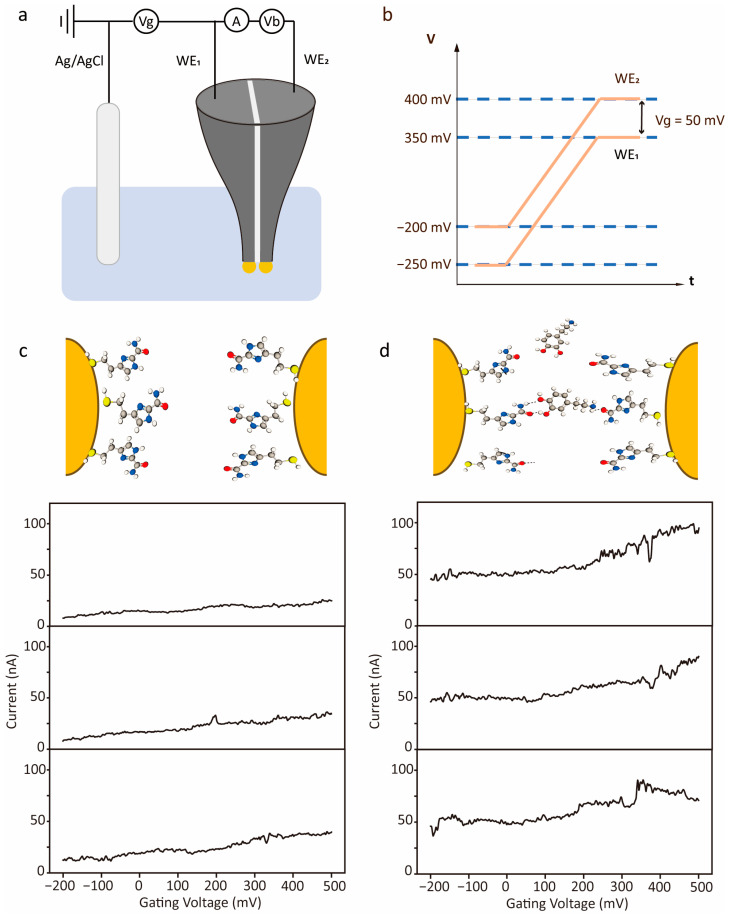
Experimental setup of electrochemical gating scanning methods and typical current–voltage response for dopamine. (**a**) Schematic illustration of the circuit of electrochemical gating. (**b**) Schematic of the potential scanning protocol for the two working electrodes. Schematic illustration of typical tunnelling current versus gating voltage response in PBS solution (**c**) and 10 μM DA solution (**d**).

The integration of a three-electrode electrochemical system (WE_1_, WE_2_, Ag/AgCl reference) was constructed (Figure 2a) to control the gating potential of the tunnelling electrodes. A constant tunnelling bias $V_{b}\;$ drove electron tunnelling, while the gate potential $V_{g}\;$(applied to WE_1_) modulated the alignment between molecular orbitals and the electrode Fermi level, enabling electrochemical control of molecular transport. As shown in Figure 2b, the potential of WE_1_ was defined as the gate potential $V_{g}$, while the surface potential of WE_2_ was set to $V_{g}+\;V_{b}$. The gate potential $V_{g}\;$ was scanned from −0.2 V to +0.4 V (vs. Ag/AgCl). This potential window covers the primary oxidation range of dopamine under neutral PBS conditions (approximately 0–0.3 V vs. Ag/AgCl, shown in Figure S1), enabling effective modulation of electrode surface potential near the redox potential of the target molecule. This range also lies within the stable electrochemical window of the gold–ICA electrode interface, avoiding significant side reactions and the oxidation of ICA that could interfere with the electrode surface status [26,27]. The 200 V/s scan rate was chosen to ensure that each scan was completed within the timescale of a single molecule diffusing into the tunnelling sensing region, thereby achieving a balance between temporal resolution and signal stability in the I–t response. Meanwhile, $V_{b}\;$ was fixed at −0.05 V to maintain weak-tunnelling response during conductance monitoring.

As shown in Figure 2c,d, the typical gate scanning curves demonstrated slightly different tunnelling current responses with and without dopamine. In the PBS background, the tunnelling current changed smoothly with only minor fluctuations. After the addition of 10 μM dopamine, pronounced current fluctuations were observed. This difference is attributed to molecular recognition at the ICA-modified electrodes. In the absence of dopamine, the current is dominated by direct tunnelling between the electrodes, while in the presence of dopamine, the molecules can be captured at the electrode surface through hydrogen bonding interactions with ICA [27,28]. This intermolecular capturing of DA molecules in the tunnelling regime alters the tunnelling pathway and modulates both orbital alignment and the stability of the DA-ICA complex, thereby converting recognition into detectable signals and enhancing the tunnelling current response.

### 3.2. Statistical Characteristics and Gating-Dependent Behavior of Dopamine

**Figure 3:**
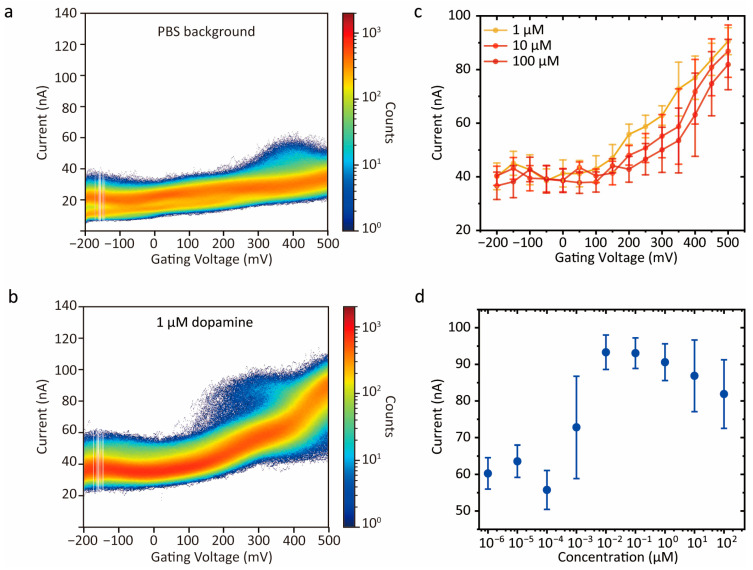
Quantification of gating-dependent scanning of DA. (**a**,**b**) Two-dimensional density map of tunnelling current (I) versus gate potential $V_{g}$ obtained in PBS solution and 1 μM dopamine solution at a scanning rate of 200 V s^−1^. Each plot was composed of approximately 20,000 gate potential scans, and the color scale indicates event counts on a logarithmic scale. (**c**) Gate-potential-dependent tunnelling current histograms of the second peak, obtained by double-Gaussian fitting, in the presence of dopamine at concentrations of 1, 10, and 100 μM, respectively. The error bar represents the standard deviation (σ) obtained from the Gaussian fitting, which accounts for the conductance dispersion resulting from varying molecule counts and stochastic conformational states. (**d**) Tunnelling current corresponding to the second peak, obtained by double-Gaussian fitting, plotted as a function of dopamine concentration at a gate potential of 500 mV vs. Ag/AgCl.

To reduce scan-to-scan variation and reveal the statistical features of the tunnelling response, approximately 20,000 scan traces were combined to generate two-dimensional current–gate potential heat maps (Figure 3a,b). Figure S2 demonstrates the scanning sweeps for 18,000 sweeps, with 2000 sweeps per plot. As shown in Figure S2, the gate-scanning data were further divided into subsets of 2000 sweeps per plot from a total of 18,000 sweeps. The consistent heat map distributions across these plots, with only minor fluctuations, demonstrate the stability and reproducibility of the device performance. In the PBS background, the current distribution appeared as a single narrow band, indicating a stable conductive background. After the addition of dopamine, the distribution broadened significantly in the gating potential scanning region and performed a bimodal feature, suggesting the dopamine-associated conductive channel. The low-conductance peak overlapped with the PBS background and was assigned to the direct tunnelling channel, whereas the high-conductance peak was attributed to a dopamine-associated transport channel formed when molecules entered the nanogap. The assignment of the second conductance peak to molecule-associated transport is supported by its absence in pure PBS, its stability over thousands of measurement cycles (Figure S3), and its systematic response to molecular concentration and species. These observations indicate that the bimodal distribution originates from discrete molecular bridging events rather than experimental artifacts such as gap fluctuations or intrinsic noise. The discrete, bimodal nature of the current distributions, combined with the transistor-like peak modulation (Figure S4), further confirms that the detected signals originate from a tunnelling-dominated transport mechanism rather than non-specific bulk electrochemical contributions. Moreover, the similar gate-dependent tunnelling responses observed at scan rates of 600 and 1200 V s^−1^ indicate that the principal response features are largely insensitive to the scan rate within this range. This suggests that the observed signal is not primarily governed by slow time-dependent processes, such as diffusion or interfacial charging (Figure S5).

The double-Gaussian fitting method is adopted from the conventional current–time (I–t) measurement system, in which the current is recorded over a long period at a fixed potential. In this work, our data processing is equivalent to collecting a large number of current signals recorded at the same fixed potential but at different time points. Therefore, Gaussian fitting can be reliably used to reflect the conductance distribution at a given potential, from which the characteristic molecular information can be further extracted.

To quantify this enhancement behavior, current histograms at different gate potentials were fitted with a bimodal Gaussian model, as shown in Figure S7. Briefly, the tunnelling current distribution was extracted from the heat map plot at a particular gating potential followed by a double-Gaussian model to identify the main tunnelling current distributions. The first tunnelling current distribution in the dopamine histogram is similar to the PBS background and was assigned as the tunnelling current background. After normalization, the relative position of the second distribution was extracted as the tunnelling enhancement signal. The gate-dependent tunnelling current response of dopamine with concentrations of 1, 10 and 100 μM is demonstrated in Figure 3c (detailed fitting of tunnelling current demonstrated in Figures S8–S10). The tunnelling current enhancement was observed when the gating potential approached the redox potential of dopamine [29]. One possible explanation is that oxidation of dopamine to a dopamine-o-quinone-like structure introduces a significantly lower LUMO (dropping from 10.22 eV in the reduced state to 8.30 eV in the oxidized state), which facilitates the electron tunnelling pathway [30]. This gate-induced alignment of molecular orbitals with the electrode Fermi level reduces the effective tunnelling barrier, thereby enhancing the conductance response. Moreover, the concentration-dependent behavior of the second-peak tunnelling current response of dopamine, obtained by bimodal fitting, as shown in Figure S11, over the concentration range from 1 pM to 100 μM at a gate potential of 500 mV is presented in Figure 3d. At this gate potential, the relative limiting current [31], quantified as a function of dopamine concentration, exhibited a sigmoidal-like trend, consistent with previous reports that the adsorption of small molecules within the tunnelling regime can be described by the Langmuir–Hill model [27,32]. These results demonstrate the characteristic gate-dependent tunnelling current response of dopamine under the present experimental conditions. To assess whether this response could be extended more generally to molecular discrimination, other small molecules were subsequently examined under the same experimental conditions.

### 3.3. Variations in Gate Responses Among Different Molecules

**Figure 4:**
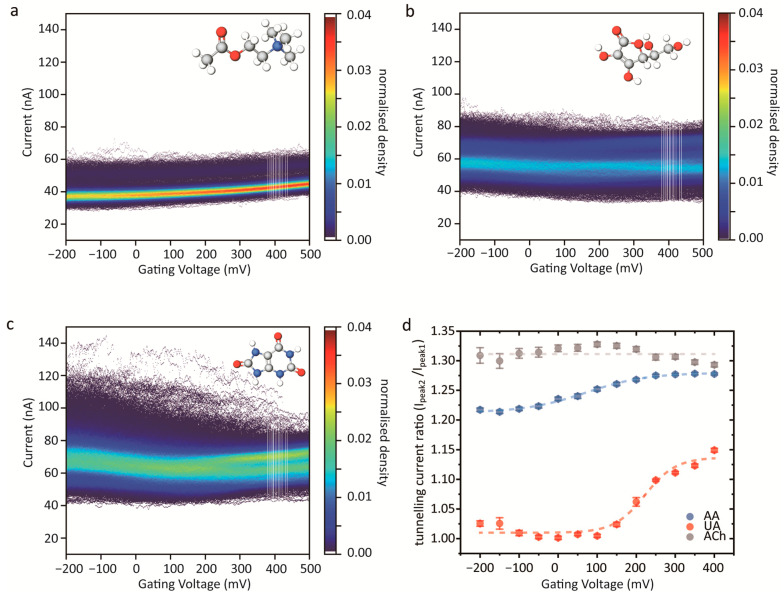
Identifying different molecules through electrochemical gating scanning methods. (**a**–**c**) Two-dimensional density maps of tunnelling currents versus $V_{g}$ measured in PBS solutions containing acetylcholine (ACh, 100 nM), ascorbic acid (AA, 100 nM), and uric acid (UA, 100 nM), respectively. (**d**) Statistical plot of the normalized tunnelling current ratio (Ipeak2/Ipeak1) as a function of gating potential for three molecules.

To validate molecular discrimination, acetylcholine (ACh), ascorbic acid (AA), and uric acid (UA) were tested under identical conditions. Figure 4a–c show typical current-gating voltage heat maps for the three molecules. As the number of recorded events differed between systems, all heat maps were normalized to allow for direct comparison.

Further quantitative analysis is presented in Figure 4d to clarify the molecular contribution to the tunnelling current response. To minimize interference from baseline fluctuations between measurements, the relative enhancement ratio $r=\frac{I_{peak\;2}}{I_{peak\;1}}$, where peak 2 and peak 1 were obtained from the double-Gaussian fitting shown in Figures S12–S15. The peak ratio values for each target molecule were extracted and plotted as a function of gate potential (Figure 4d), indicating that the second peak was largely independent of gate potential. As ACh is a non-redox-active molecule under the present conditions, the two tunnelling current distributions are interpreted as two relatively stable conductance states of the ACh-associated junction. In contrast, AA demonstrated a Boltzmann-like trend [32,33], which indicates that the AA was oxidized at the half-response gate potential $V_{1/2}=70.9\;\pm \;9.1\;$ mV, suggesting a gate-dependent transition consistent with AA oxidation. This value is in reasonable agreement with the oxidation peak observed in the CV response of the nanoelectrode [34]. This transition is strongly supported by recent computational studies indicating that the HOMO-LUMO gap (ΔE) of AA narrows dramatically from 3.64 eV to 1.52 eV upon oxidation to dehydroascorbic acid (DHA) [35]. Such a substantial reduction in ΔE provides a clear physical basis for the observed gate-dependent conductance enhancement, confirming the link between electrochemical gating and molecular transport. For UA, clear splitting of the tunnelling current distribution was observed from approximately 200 mV vs. Ag/AgCl, and the fitted current ratio response gave $V_{1/2}=222\;\pm \;13.8\;$ mV. According to previous work [36,37,38], uric acid oxidation is activated from around 200 mV and typically shows an oxidation peak at 300–400 mV vs. Ag/AgCl under near-neutral conditions. Together, these molecule-dependent gate response patterns suggest that electrochemical gating can provide an additional statistical dimension for distinguishing molecules with similar structures, with potential value for future single-molecule detection in solution.

## 4. Discussion

The results of this study are consistent with previous reports on electrochemical gate modulation of molecular-scale conductance. Xiao et al. reported that electrochemical gates modulate molecular-scale conductance by altering molecular energy level alignment [22], while Li et al. demonstrated that interfacial potential strongly influences molecular transport mechanisms [23]. In contrast to these earlier studies, which mainly focused on conductance modulation, this work applies gate-dependent conductance distribution as a feature for molecular identification. This response can serve as an electrical fingerprint for distinguishing structurally similar molecules in complex chemical systems and extends electrochemically gated tunnelling from conductance measurement to molecular analysis.

While the molecules investigated here possess distinct redox potential, the gate-dependent response provides a multi-dimensional electronic signature by integrating orbital alignment and junction coupling. This approach expands molecular recognition beyond simple potential-based detection. Future studies on isomers or molecules with overlapping redox profiles will further evaluate the resolution limits of this fingerprinting method.

Despite these advances, several limitations remain. Firstly, only a small number of molecules have been examined so far. Future studies involving more structurally similar molecules (e.g., different catecholamines) will be needed to further test the discrimination ability of this method [17]. In addition, the mechanisms underlying the gate response behaviors of different molecules require further investigation. Combining experimental measurements with theoretical calculations may provide a clearer understanding of the mechanism [39]. Thirdly, further improvements in instrumentation and data analysis may also improve performance in complex samples [40].

## 5. Conclusions

In this work, recognition tunnelling was combined with electrochemical gating for molecular-scale identification. By introducing a tunable gate potential, a conventional two-electrode tunnelling platform was converted into a three-electrode electrochemical system. Scanning the gate potential under a constant tunnelling bias changed the alignment between molecular orbitals and the electrode Fermi level, leading to gate-dependent changes in molecular transport. When target molecules entered the tunnelling region, the current distribution showed a bimodal structure, with one peak from the background signal and another from molecule-related conduction. Comparative analysis of ascorbic acid, acetylcholine, and uric acid showed distinct gate-dependent conductance responses, forming characteristic electrochemical fingerprints. This work provides a useful method for improving label-free molecular-scale identification and distinguishing small molecules in complex chemical systems.

## Data Availability

The data that support the findings of this study are available from the corresponding authors upon reasonable request.

## Supplementary Materials

The following supporting information can be downloaded at: https://www.mdpi.com/article/10.3390/s26092896/s1, Figure S1: CV plot of dopamine recorded on the tunnelling nanoelectrode. Seven CV curves were sequentially recorded with the following order: PBS background before (−0.5 to 0.6 V), DA 0.2 V/s (−0.5 to 0.2 V), DA 0.3 V/s (−0.5 to 0.3 V), DA 0.4 V/s (−0.5 to 0.4 V), DA 0.5 V/s (−0.5 to 0.5 V), DA (−0.5 to 0.6 V), and PBS background after (−0.5 to 0.6 V). The gradual extension of the upper potential limit allows the oxidation behavior of dopamine to be identified while minimizing possible electrode fouling. An oxidation current emerges when the potential exceeds approximately 0 V (vs. Ag/AgCl); Figure S2: Gating-dependent current-voltage scanning of tunnelling junctions in PBS solution (10 mM, pH = 7.4) from 1–18,000 sweeps. Each heat map plot shows 2000 continuous sweeps with gating voltage scanning from −200 mV to +400 mV; Figure S3: Current histogram at Vg = 400 mV in DA solution from 1–22,500 sweeps. Each histogram shows 2500 continuous sweeps; Figure S4: Plot represents transistor-like tunnelling current enhancement modulation at 1 μM concentration. The plot illustrates the current ratio obtained by dividing the signal-containing sweeps by the pure background sweeps as a function of gate potential (V_g_). A clear peak-shaped trajectory is observed, demonstrating the transistor-like modulation of the molecular tunnelling junction under electrochemical gating; Figure S5: Gating-dependent scanning of tunnelling junction in 10 mM PBS background and 10 μM dopamine solution (diluted by 10 mM PBS solution) under scanning rate of 600 V/s and 1200 V/s, respectively; Figure S6: Gating-dependent scanning of 10 mM PBS (upper, pH = 7.4) and 1 μM dopamine (below, diluted in 10 mM PBS solution) under scanning rate of 200 V/s on three different ICA-functionalised devices; Figure S7: Illustration of the conversion of the heat map into tunnelling current distribution histograms and subsequent double-Gaussian fitting. The analysis shown here was performed using the heat map obtained for a 1 μM dopamine solution; Figure S8: Current distributions of 1 µM dopamine at different gate potentials and corresponding double-Gaussian fitting, where Peak 1 (orange dashed line) represents the dominant background tunnelling current component and Peak 2 (yellow dashed line) corresponds to the higher-current component associated with molecular contributions; Figure S9: Current distributions of 10 µM dopamine at different gate potentials and corresponding double-Gaussian fitting, where Peak 1 (orange dashed line) represents the dominant background tunnelling current component and Peak 2 (yellow dashed line) corresponds to the higher-current component associated with molecular contributions; Figure S10: Current distributions of 100 µM dopamine at different gate potentials and corresponding double-Gaussian fitting, where Peak 1 (orange dashed line) represents the dominant background tunnelling current component and Peak 2 (yellow dashed line) corresponds to the higher-current component associated with molecular contributions; Figure S11: Current distributions and bimodal fitting of dopamine from 1 pM to 100 µM at V_g_ = 500 mV vs. Ag/AgCl, V_b_ = 100 mV, where Peak 1 (orange dashed line) represents the dominant background tunnelling current component and Peak 2 (yellow dashed line) corresponds to the higher-current component associated with molecular contributions; Figure S12: Current distributions of ascorbic acid at different gate potentials and corresponding double-Gaussian fitting. Histograms of tunnelling current recorded for ascorbic acid (AA) at gate potentials ranging from −200 mV to 400 mV with a step of 50 mV. Each distribution was fitted using a double-Gaussian model (blue line), where Peak 1 (orange dashed line) represents the dominant background tunnelling current component and Peak 2 (green dashed line) corresponds to the higher-current component associated with molecular contributions; Figure S13: Current distributions of acetylcholine at different gate potentials and corresponding double-Gaussian fitting. Histograms of tunnelling current recorded for acetylcholine (ACh) at gate potentials ranging from −200 mV to 400 mV with a step of 50 mV. Each distribution was fitted using a double-Gaussian model (blue line), where Peak 1 (orange dashed line) represents the dominant background tunnelling current component and Peak 2 (green dashed line) corresponds to the higher-current component associated with molecular contributions; Figure S14. Enlarged view of acetylcholine current distributions highlighting the second peak; Figure S15: Current distributions of uric acid at different gate potentials with double-Gaussian fitting. Histograms of tunnelling current recorded for uric acid (UA) at gate potentials ranging from −200 mV to 400 mV with a step of 50 mV. The current distributions were fitted using a double-Gaussian model (blue line), where Peak 1 (orange dashed line) represents the dominant background tunnelling current component and Peak 2 (green dashed line) corresponds to the higher-current component associated with molecular contributions.
