# Supplementary material for: Fingerprint Recognition Based on Molecular-Scale Conductance Response via Electrochemically Gated Quantum Tunnelling
Source: Sensors (Basel). 2026 May 5;26(9):2896. doi: 10.3390/s26092896 (PMC13165982; doi:10.3390/s26092896)
Supplement: Supplementary file 1 [file sensors-26-02896-s001.zip › sensors-4230047-supplementary.pdf]

Supporting Information

Fingerprint Recognition Based on Molecule-Scale Conductance Response via Electrochemically Gated Quantum Tunnelling

Zifan Wang [1], Long Yi [1,2]*, Ga Zhang [1], Xufei Ma [1], Ye Tian,[3] Bintian Zhang [4], Xu Liu,[1] and Longhua Tang [1,2]*

[1] State Key Laboratory of Extreme Photonics and Instrumentation, Interdisciplinary Center for Quantum Information, College of Optical Science and Engineering, Zhejiang University, Hangzhou 310027, China

[2] Nanhu Brain-Computer Interface Institute; Second Affiliated Hospital School of Medicine, Hangzhou, Zhejiang, 311100, China

[3] State Key Laboratory of Fluid Power and Mechatronic Systems, College of Mechanical Engineering, Zhejiang University, Hangzhou 310058, China

[4] Shenzhen Key Laboratory of Precision Measurement and Early Warning Technology for Urban Environmental Health Risks, School of Environmental Science and Engineering, Southern University of Science and Technology, Shenzhen 518055, China

* Correspondence: long.yi@zju.edu.cn (YL), lhtang@zju.edu.cn (LT)

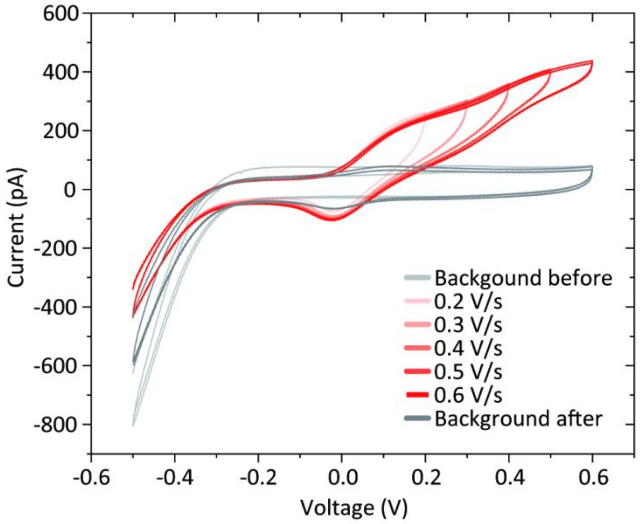

Figure S1. CV plot of dopamine recorded on the tunnelling nanoelectrode. Seven CV curves were sequentially recorded with the following order: PBS background before (−0.5 to 0.6 V), DA 0.2 V /s (−0.5 to 0.2 V), DA 0.3 V /s (−0.5 to 0.3 V), DA 0.4 V /s (−0.5 to 0.4 V), DA 0.5 V /s (−0.5 to 0.5 V), DA (−0.5 to 0.6 V), and PBS background after (−0.5 to 0.6 V). The gradual extension of the upper potential limit allows the oxidation behavior of dopamine to be identified while minimizing possible electrode fouling. An oxidation current emerges when the potential exceeds approximately 0 V (vs Ag/AgCl).

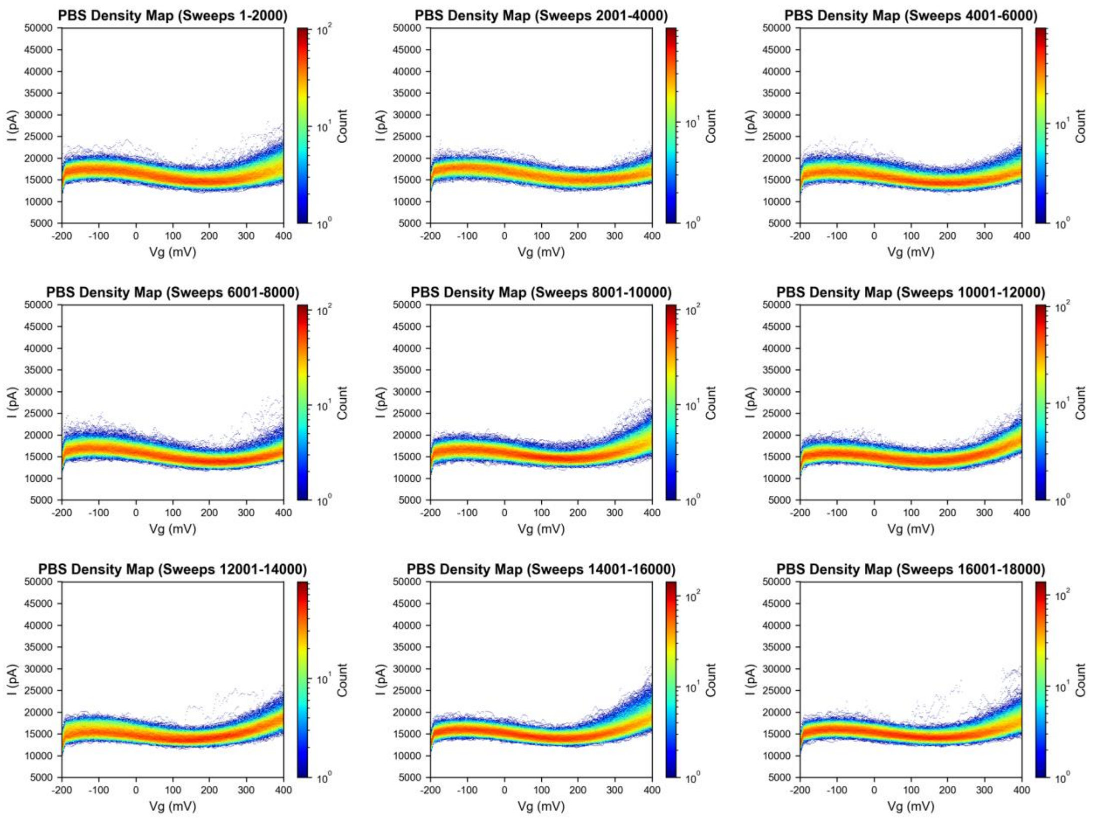

Figure S2. Gating-dependent current-voltage scanning of tunnelling junctions in PBS solution (10 mM, pH=7.4) from 1-18000 sweeps. Each heat map plot shows 2000 continuous sweeps with gating voltage scanning from -200 mV to +400 mV.

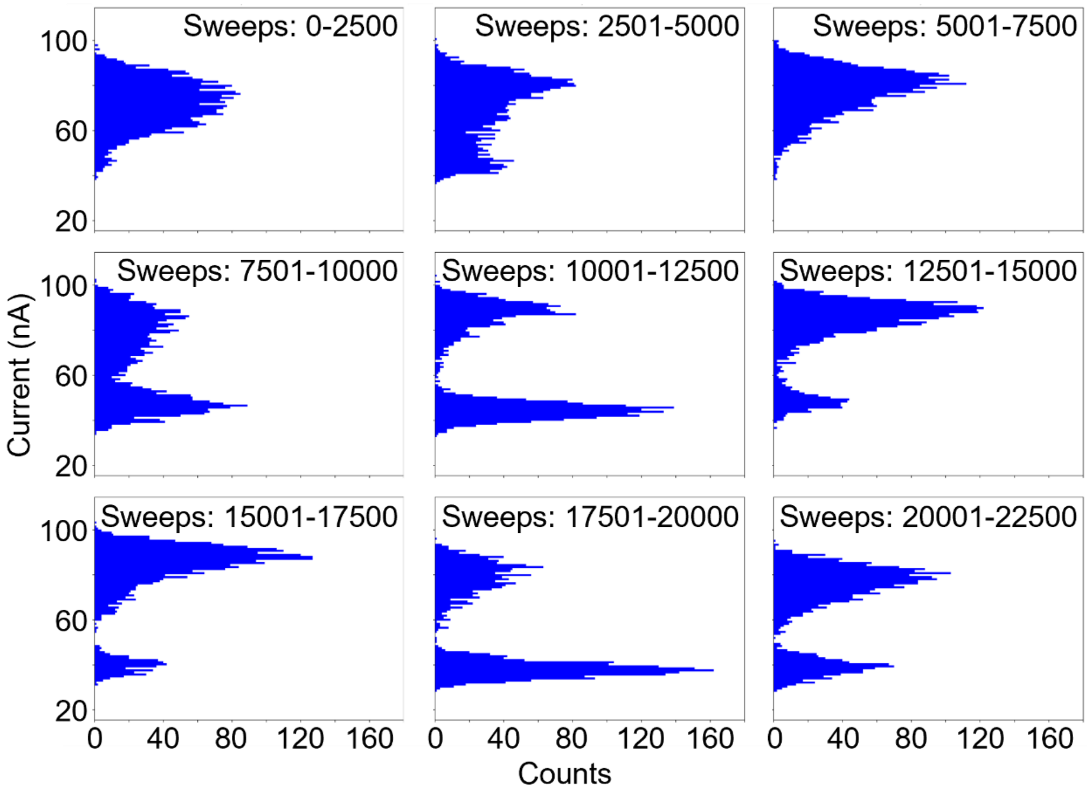

Figure S3. Current histogram at Vg = 400 mV in DA solution from 1-22500 sweeps. Each histogram shows 2500 continuous sweeps.

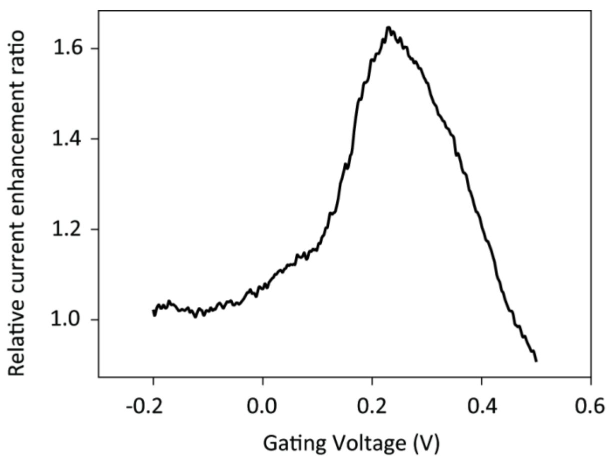

Figure S4. Plot represents transistor-like tunnelling current enhancement modulation at 1 μM concentration. The plot illustrates the current ratio obtained by dividing the signal-containing sweeps by the pure background sweeps as a function of gate potential (V_g). A clear peak-shaped trajectory is observed, demonstrating the transistor-like modulation of the molecular tunnelling junction under electrochemical gating.

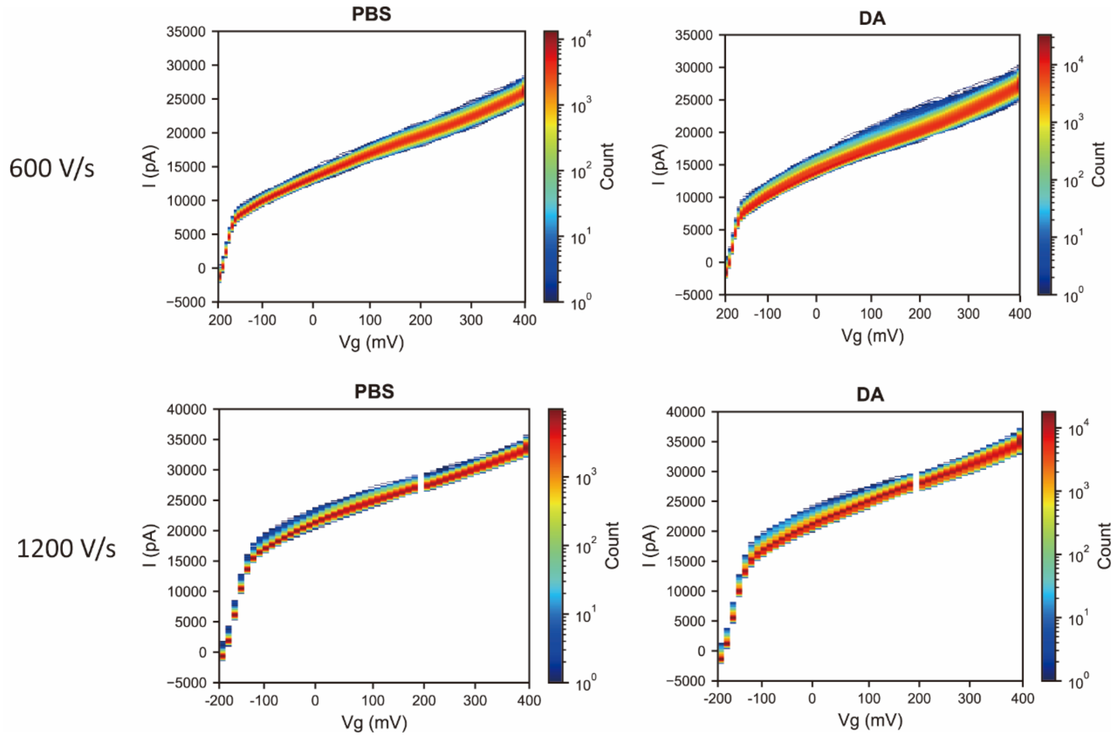

Figure S5. Gating-dependent scanning of tunnelling junction in 10 mM PBS background and 10 μM dopamine solution (diluted by 10 mM PBS solution) under scanning rate of 600 V/s and 1200 V/s, respectively.

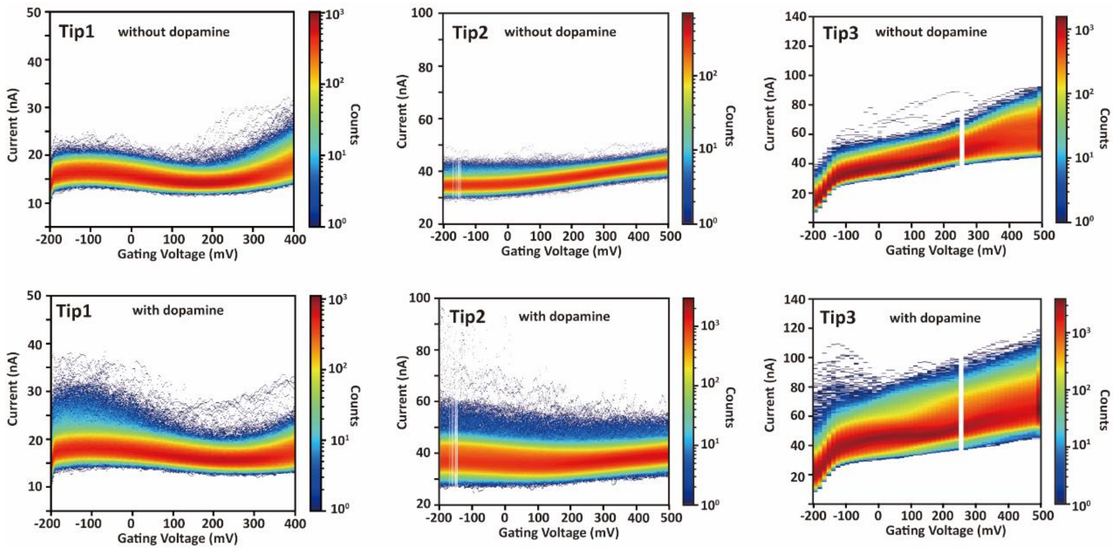

Figure S6. Gating-dependent scanning of 10 mM PBS (upper, pH = 7.4) and 1 μM dopamine (below, diluted in 10 mM PBS solution) under scanning rate of 200 V/s on three different ICA-functionalised devices.

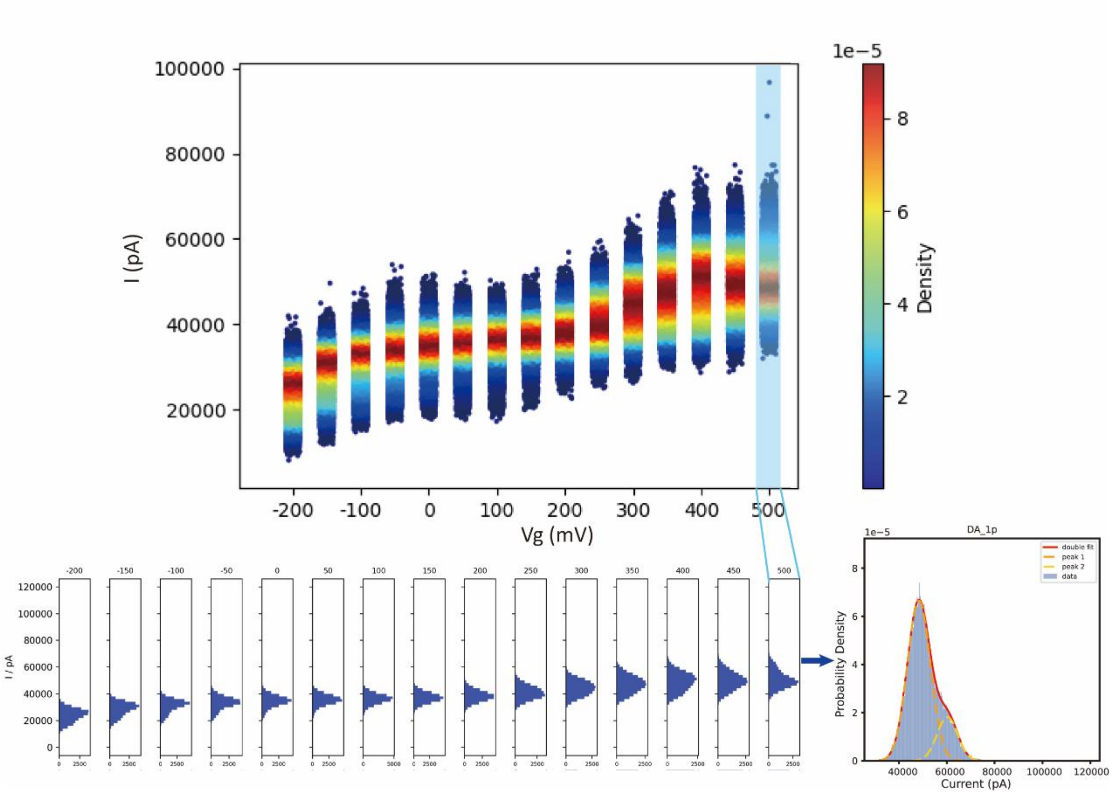

Figure S7. Illustration of the conversion of the heat map into tunnelling current distribution histograms and subsequent double-Gaussian fitting. The analysis shown here was performed using the heat map obtained for a 1 μM dopamine solution.

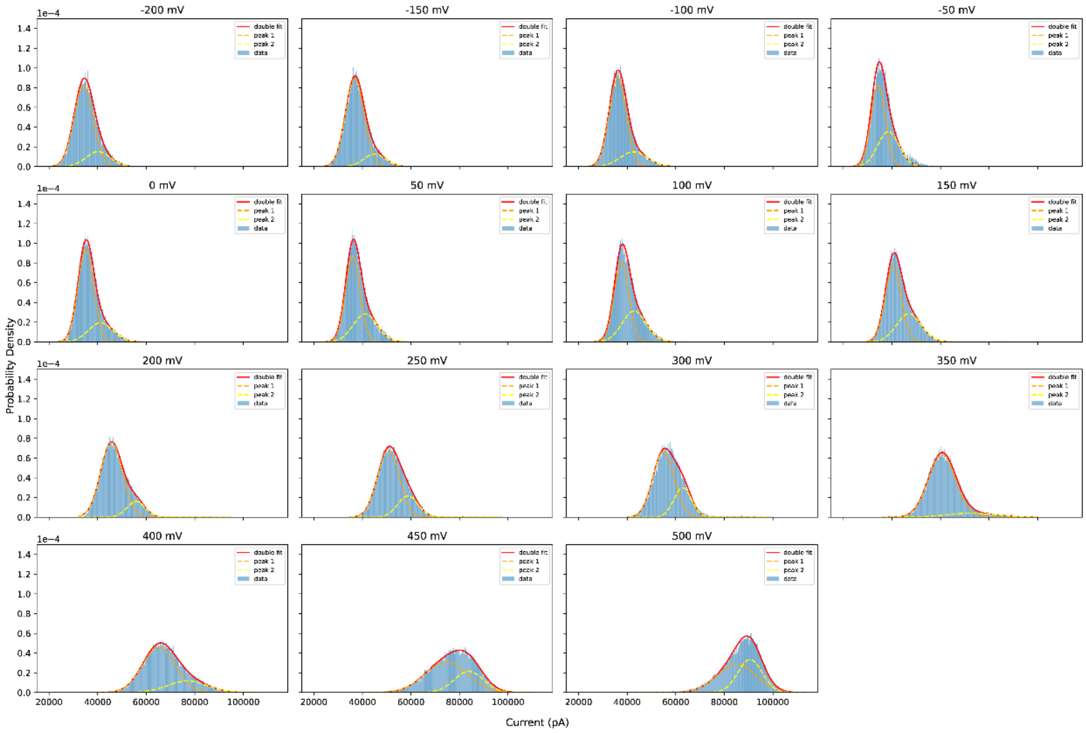

Figure S8. Current distributions of 1 μM dopamine at different gate potentials and corresponding double-Gaussian fitting, where Peak 1 (orange dashed line) represents the dominant background tunnelling current component and Peak 2 (yellow dashed line) corresponds to the higher-current component associated with molecular contributions.

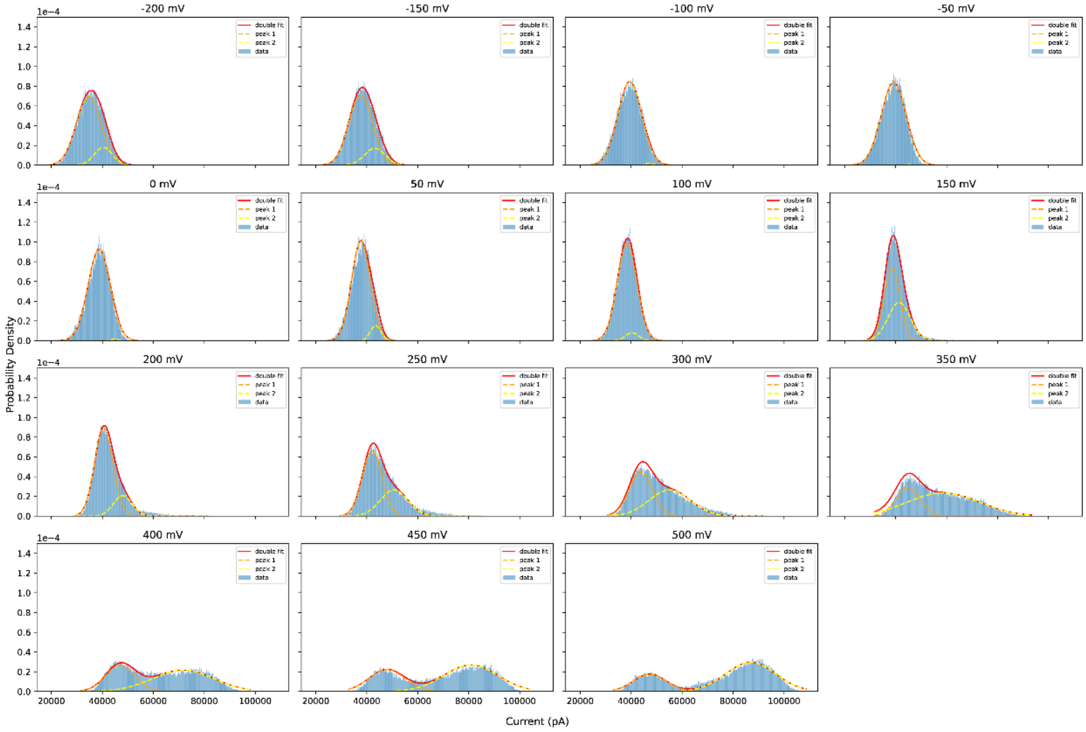

Figure S9. Current distributions of 10 μM dopamine at different gate potentials and corresponding double-Gaussian fitting, where Peak 1 (orange dashed line) represents the dominant background tunnelling current component and Peak 2 (yellow dashed line) corresponds to the higher-current component associated with molecular contributions.

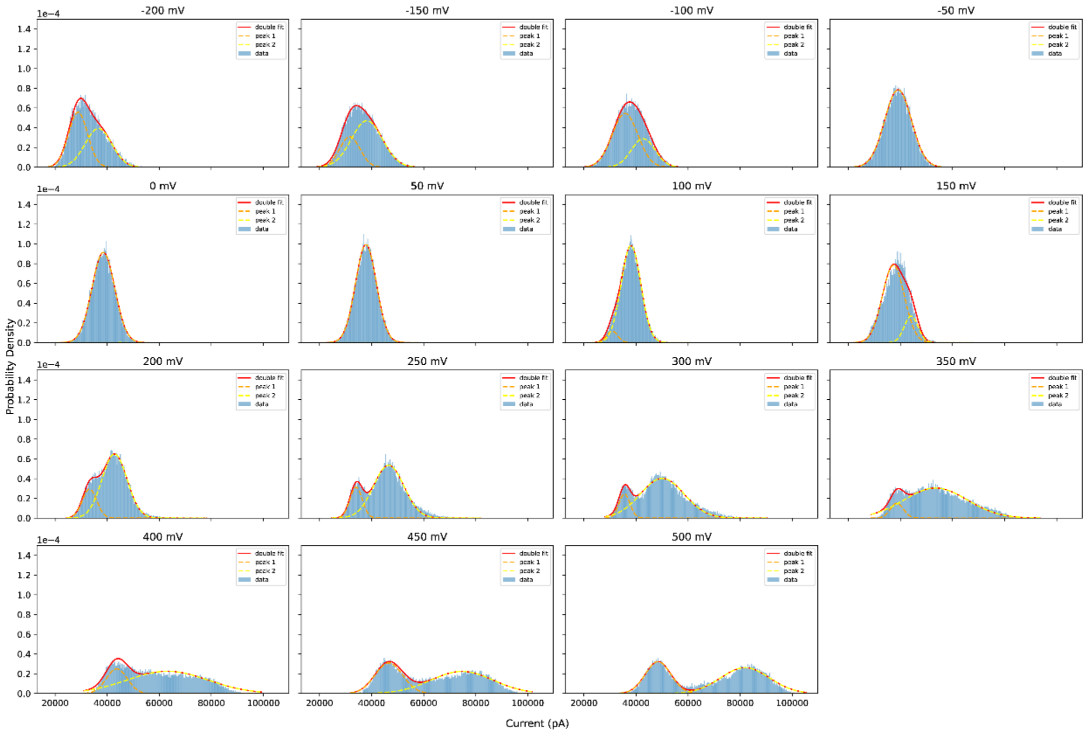

Figure S10. Current distributions of 100 μM dopamine at different gate potentials and corresponding double-Gaussian fitting, where Peak 1 (orange dashed line) represents the dominant background tunnelling current component and Peak 2 (yellow dashed line) corresponds to the higher-current component associated with molecular contributions.

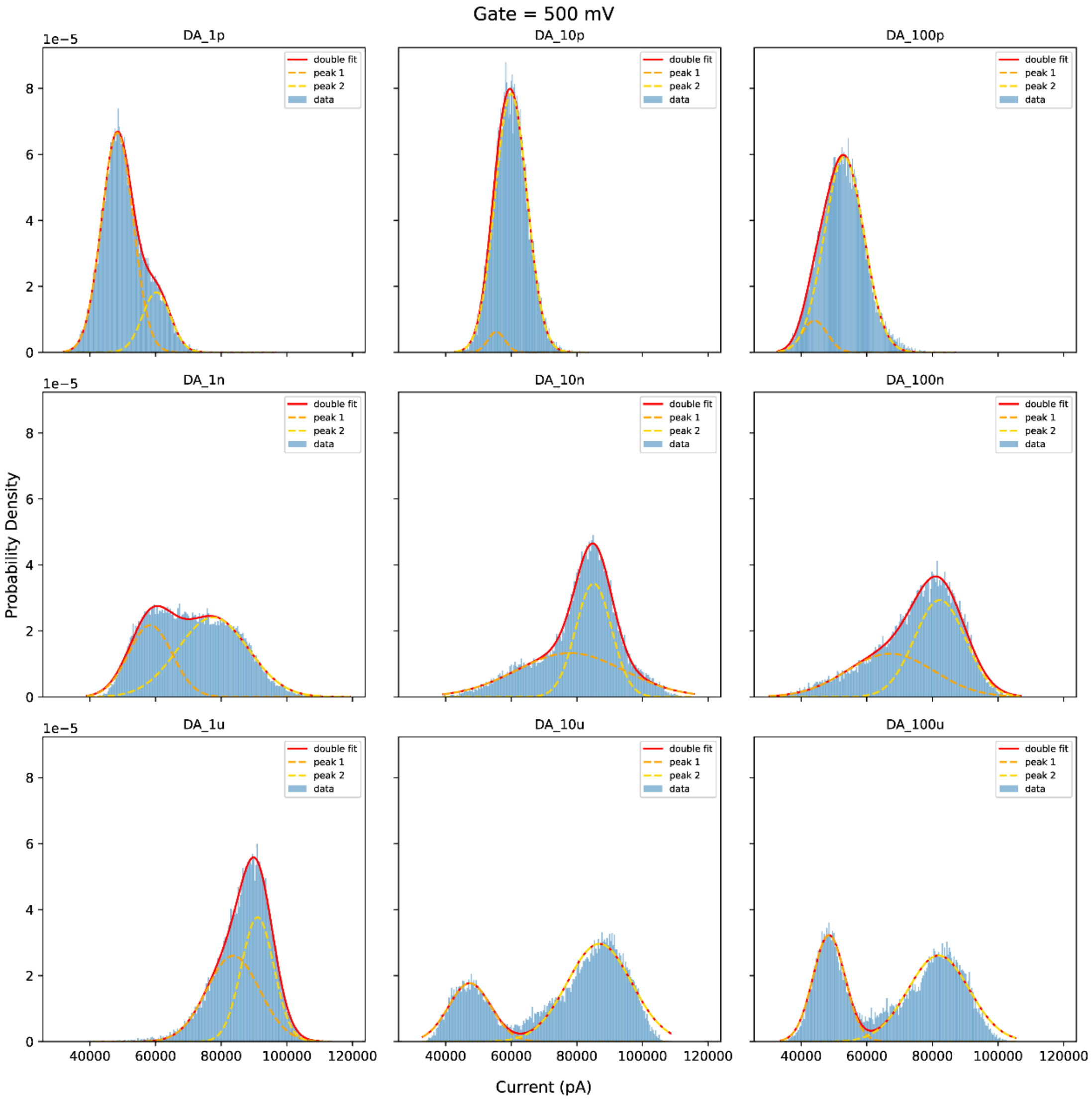

Figure S11. Current distributions and bimodal fitting of dopamine from 1 pM to 100 µM at V_g = 500 mV vs Ag/AgCl, V_b = 100 mV, where Peak 1 (orange dashed line) represents the dominant background tunnelling current component and Peak 2 (yellow dashed line) corresponds to the higher-current component associated with molecular contributions.

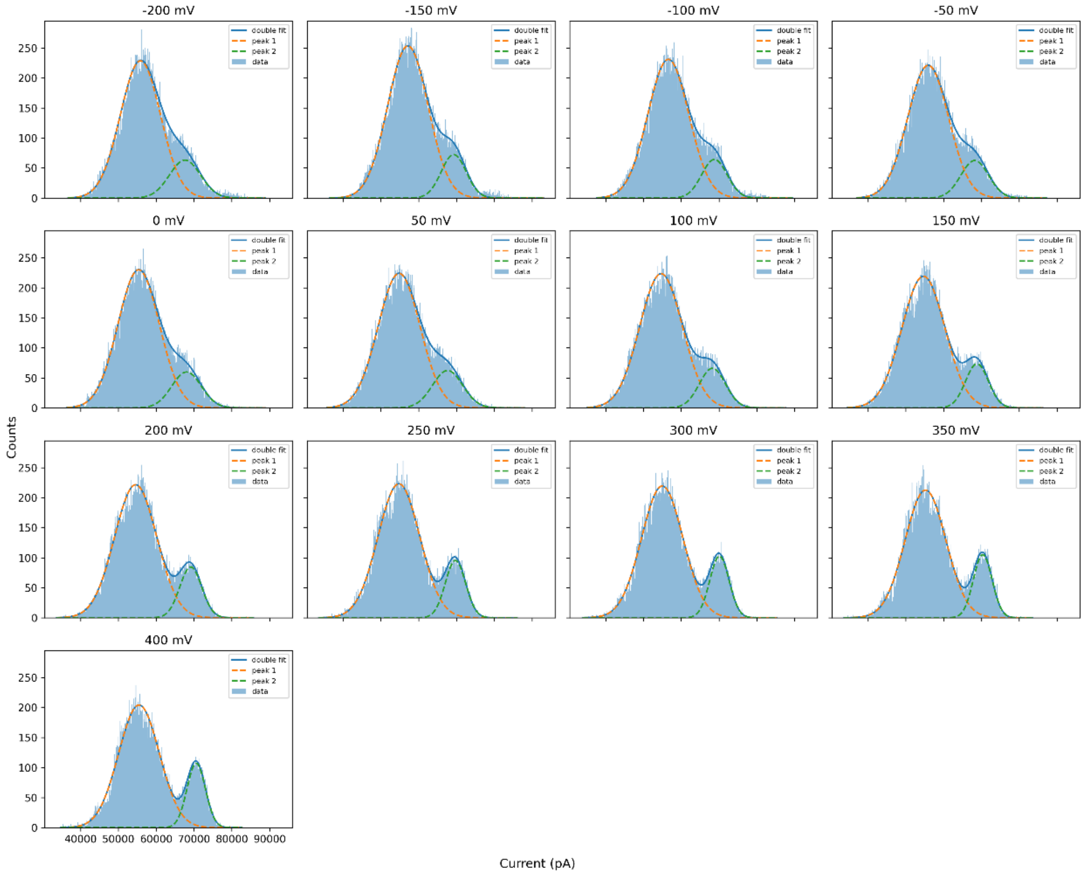

Figure S12. Current distributions of ascorbic acid at different gate potentials and corresponding double-Gaussian fitting. Histograms of tunnelling current recorded for ascorbic acid (AA) at gate potentials ranging from −200 mV to 400 mV with a step of 50 mV. Each distribution was fitted using a double-Gaussian model (blue line), where Peak 1 (orange dashed line) represents the dominant background tunnelling current component and Peak 2 (green dashed line) corresponds to the higher-current component associated with molecular contributions.

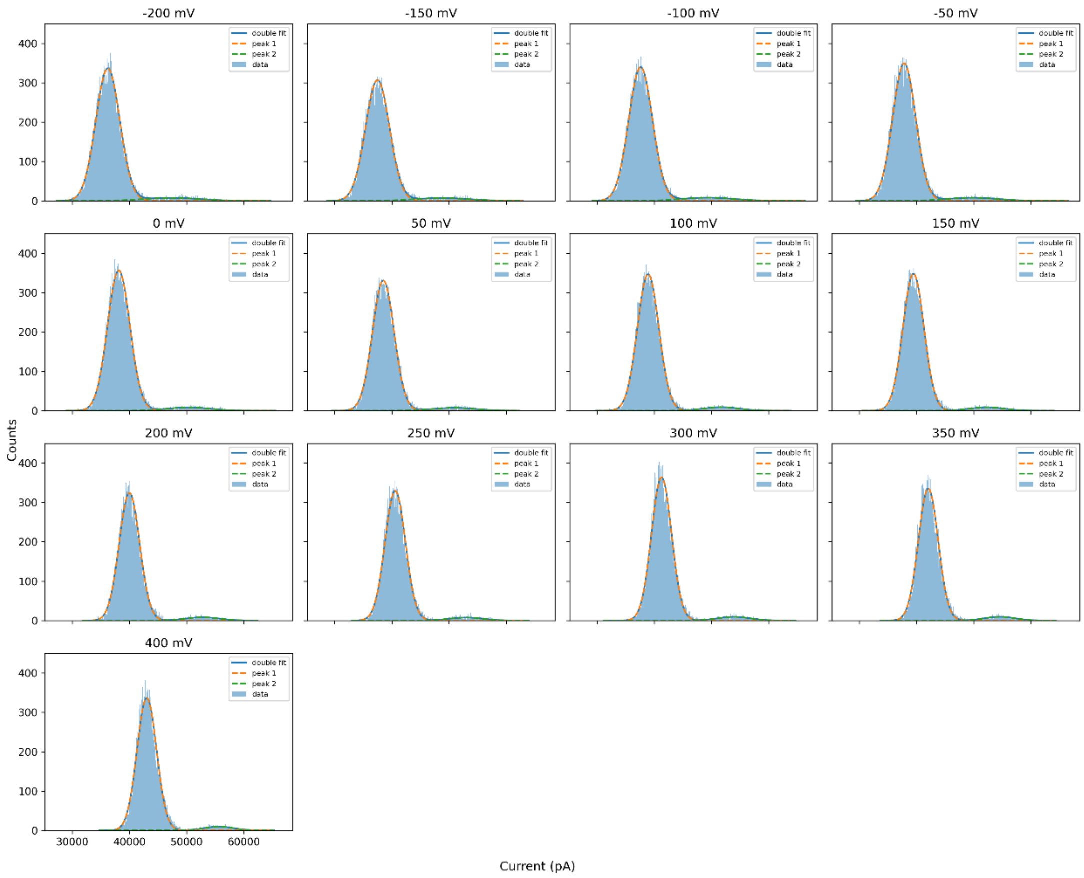

Figure S13. Current distributions of acetylcholine at different gate potentials and corresponding double-Gaussian fitting. Histograms of tunnelling current recorded for acetylcholine (ACh) at gate potentials ranging from −200 mV to 400 mV with a step of 50 mV. Each distribution was fitted using a double-Gaussian model (blue line), where Peak 1 (orange dashed line) represents the dominant background tunnelling current component and Peak 2 (green dashed line) corresponds to the higher-current component associated with molecular contributions.

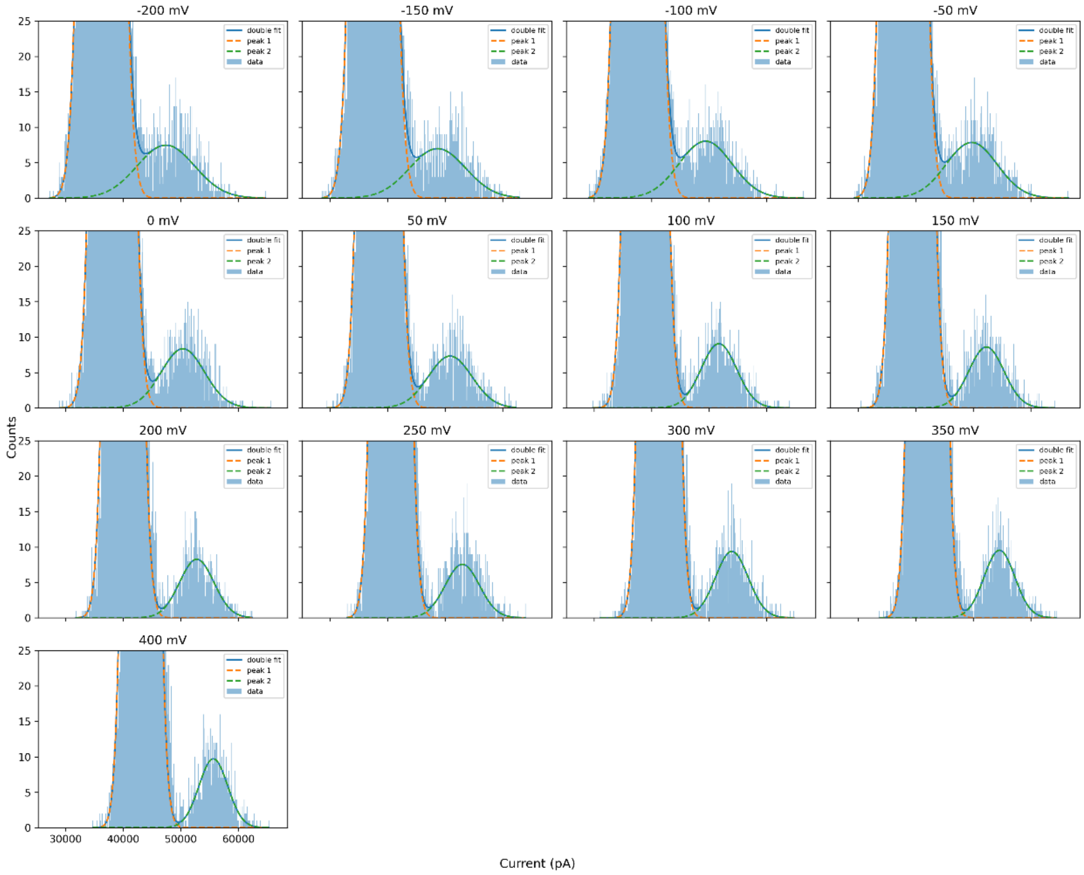

Figure S14. Enlarged view of acetylcholine current distributions highlighting the second peak.

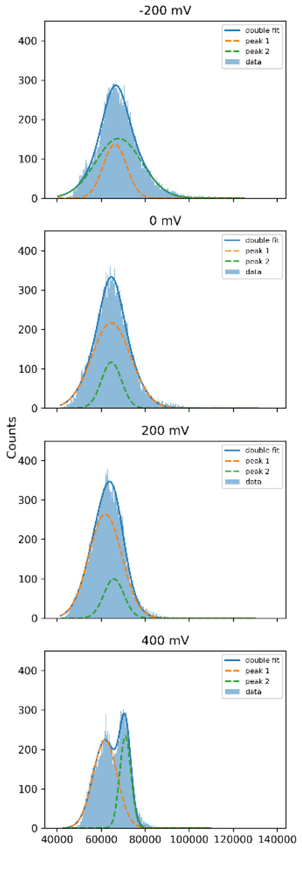

Figure S15. Current distributions of uric acid at different gate potentials with double-Gaussian fitting. Histograms of tunnelling current recorded for uric acid (UA) at gate potentials ranging from −200 mV to 400 mV with a step of 50 mV. The current distributions were fitted using a double-Gaussian model (blue line), where Peak 1 (orange dashed line) represents the dominant background tunnelling current component and Peak 2 (green dashed line) corresponds to the higher-current component associated with molecular contributions.
